# Inverse Doppler Effects in Broadband Acoustic Metamaterials

**DOI:** 10.1038/srep32388

**Published:** 2016-08-31

**Authors:** S. L. Zhai, X. P. Zhao, S. Liu, F. L. Shen, L. L. Li, C. R. Luo

**Affiliations:** 1 Smart Materials Laboratory, Department of Applied Physics, Northwestern Polytechnical University, Xi’an, 710129, P.R. China

## Abstract

The Doppler effect refers to the change in frequency of a wave source as a consequence of the relative motion between the source and an observer. Veselago theoretically predicted that materials with negative refractions can induce inverse Doppler effects. With the development of metamaterials, inverse Doppler effects have been extensively investigated. However, the ideal material parameters prescribed by these metamaterial design approaches are complex and also challenging to obtain experimentally. Here, we demonstrated a method of designing and experimentally characterising arbitrary broadband acoustic metamaterials. These omni-directional, double-negative, acoustic metamaterials are constructed with ‘flute-like’ acoustic meta-cluster sets with seven double meta-molecules; these metamaterials also overcome the limitations of broadband negative bulk modulus and mass density to provide a region of negative refraction and inverse Doppler effects. It was also shown that inverse Doppler effects can be detected in a flute, which has been popular for thousands of years in Asia and Europe.

Sound waves are a fundamental vibration mode in nature. The Doppler effect refers to a change in frequency of a wave source as a consequence of the relative motion between the wave source and an observer^1^,^2^. This phenomenon is applied to several fields, including scientific research, space technology, traffic management and medical diagnosis^3^,^4^,^5^,^6^. In 1968, Veselago^7^ theoretically predicted that metamaterials^8^,^9^,^10^ with negative refractions^11^,^12^ can induce the inverse Doppler effect. An inverse Doppler shift in electromagnetic waves can be achieved by using a transmission line^13^,^14^,^15^, with backward dipolar spin waves in a magnetic thin film^16^,^17^ and photonic crystals^18^,^19^; in acoustics, a phononic crystal^20^ is a material commonly used to obtain the inverse Doppler effect. However, this abnormal phenomenon appears experimentally only in the frequency range corresponding to the energy gap, and the band is narrow. Based on non-resonant elements, a quasi-1D, double-negative acoustic metamaterial can also be used to generate the inverse Doppler effect in a low-frequency region^21^. Some projects employ nested elements, such as concentric square rings, to achieve broadband absorption and to generate a double- and even triple-band absorption^22^,^23^. Non-resonant metamaterial elements can also be used for broadband cloaks^24^,^25^. Nevertheless, a general method of designing and fabricating a controllable broadband, double-negative acoustic metamaterial has yet to be reported; an arbitrary broad-band acoustic transmission and inverse Doppler effect are always difficult things.

The mechanism of metamaterials is based on resonance in periodic structures, such as electromagnetic metamaterials with metallic wires exhibiting negative permittivity or with split resonance rings displaying negative magnetic permeability^26^,^27^ and acoustic metamaterials with a high-density solid core and a soft coating exhibiting a negative dynamic mass density^28^ or subwavelength Helmholtz resonators with a negative effective dynamic bulk modulus^29^. The bandwidth of this resonance is narrow in nature. Previous theories^30^,^31^ and experiments^32^,^33^ demonstrated that a metamaterial in each distinct unit cell resonates at its inherent frequency and elicits almost no coupling effect with other materials; thus, a weak interaction system can be formulated. The total response of a weak interaction system can be treated as an overlap of the single resonance spectrum of each type of unit cell. Multiband or broadband metamaterials can be developed using a simple method because of this intriguing feature. Moreover, in our previous work^34^, we have demonstrated the feasibility to realise broadband negative mass density by using a meta-atom cluster model.

In this paper, inspired by the fact that visible light comprises seven light colours and musical temperament consists of seven musical scales, a ‘flute-like’ model of an acoustic meta-cluster, containing seven meta-molecules with different dimensions was proposed. A metamaterial sample of a double meta-cluster was experimentally constructed and transmission and reflection results were obtained through experimental measurements and numerical simulations, from which mass density and bulk modulus were derived to be simultaneously negative in a broadband range. Using this broadband double-negative sample, we experimentally measured refractions and inverse Doppler effects from 1.186 kHz to 6.534 kHz. It was also shown that inverse Doppler effects can be detected in a flute, which has been popular for thousands of years in Asia and Europe.

## Results

**Figure 1:**
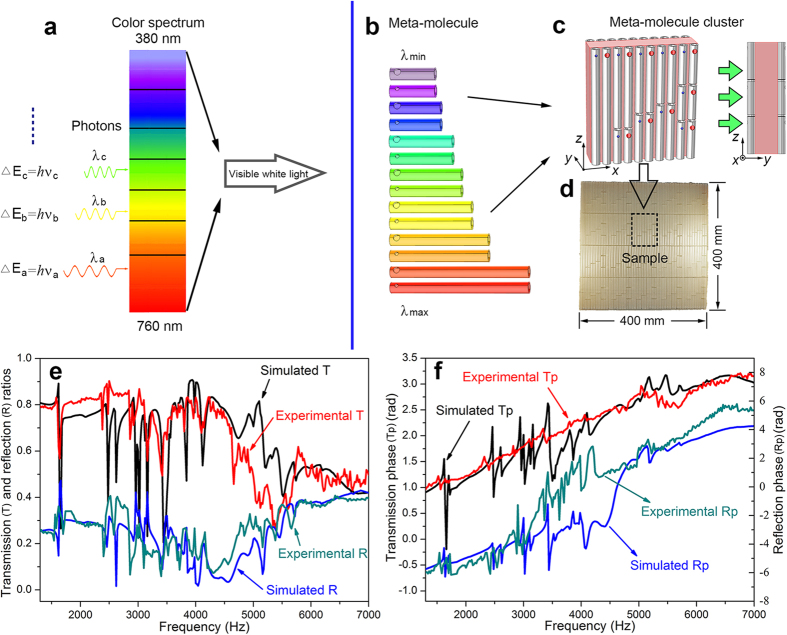
Model and behaviour of the acoustic meta-molecule cluster. (**a**) Relationship between rainbow and visible white light. The frequency of a photon is determined by its energy, and lights with different frequencies have different colours. When the seven light colours are mixed, visible white light appears. (**b**), The meta-molecule units, constructed with different dimensions, generate acoustic surface plasmon polaritons with various frequencies and anomalous acoustic properties. Seven meta-molecules are combined to form a cluster; in a cluster, every meta-molecule contains two fine structures to obtain broadband abnormal acoustic properties. (**c**) A schematic and a lateral view of the meta-molecule cluster. Sound waves are propagated along the positive direction of the *Y*-axis. (**d**) A photograph of the meta-molecule cluster sample. The dimensions of the sample are *x* × *z* = 400 mm × 400 mm. (**e**) Transmitted and reflected ratios of the metamaterial sample as a function of frequency. (**f**) Transmitted and reflected phases of the sample. The black and red lines indicate the simulated and experimental transmittance curves, respectively; the blue and purple lines represent the simulated and experimental reflectance curves, respectively.

It is known that electromagnetic waves consist of several photons with different energies and frequencies; visible white light (400–800 THz) can be obtained by mixing disparate photons with colours corresponding to red, orange, yellow, green, cyan, blue and purple. Analogous to photons, surface plasmon polaritons generated by the resonance of a meta-molecule^32^ are found in acoustics; the frequency of plasmon polaritons is related to the geometry of structural units. Nevertheless, the frequency bandwidth is relatively narrow^35^,^36^. The analogy between acoustic surface plasmon polaritons and photons is shown in Fig. 1. The frequency *ν* of a photon is determined by its energy *ε*, which is expressed as follows:


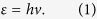


No interactions occur among photons. Acoustic metamaterials can generate oscillations of surface plasmon polaritons near the resonant frequency *ω*_0_ (from *ω*_0_ − *δω* to *ω*_0_ + *δω*); these oscillations directly produce abnormal effective parameters of materials, with mass density *ρ*_*eff*_ and bulk modulus *E*_*eff*_ 35:


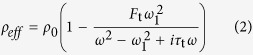


and


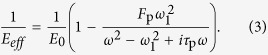


Experiments and theories have demonstrated the lack of interactions among surface plasmon polaritons^30^,^31^,^32^,^33^. Acoustic artificial meta-molecules can simultaneously produce negative mass density and bulk modulus^35^; as such, a meta-molecule cluster is formed by combining meta-molecules with different resonant frequencies in a particular arrangement.

One meta-molecule cluster is composed of *j* types of meta-molecules, where *j* = 1, 2, …, *n*. Each meta-molecule can generate a double-negative frequency range near the resonant frequency *ω*_*j*0_ such that 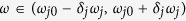, where 
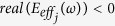
 and 
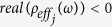
. Therefore, the effective mass density *ρ*_*effc*_ (*ω*) and the effective bulk modulus *E*_*effc*_ (*ω*) of this meta-molecule cluster can be expressed as follows:


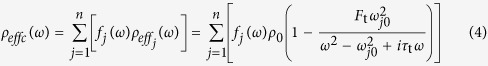


and


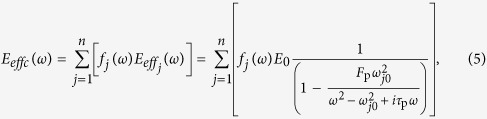


where *f*_*j*_ (*ω*) is the distribution density function of the *j*th meta-molecule at the frequency of *ω*. If *n* is sufficiently large, the double-negative frequency ranges of adjacent meta-molecules overlap; thus, the mass density and bulk modulus of this meta-molecule cluster are simultaneously negative in a broadband frequency range.

The resonant frequency *ω*_0_ is determined based on the tube length and diameter of the side hole. Inspired by the fact that visible light comprises seven light colours and audible sound can be divided into seven musical scales (1, 2, 3, 4, 5, 6 and 7) and several tones (A, B, C, D and so on), we construct the general broadband acoustic double-negative metamaterials by using a meta-molecule cluster set. The effective mass density *ρ*_*effs*_ (*ω*) and bulk modulus *E*_*effs*_ (*ω*) can be expressed as follows:


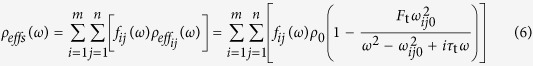


and


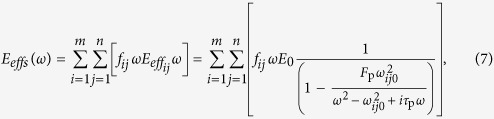


where *i* is the number of the meta-molecule clusters (*i* = 1, 2, …, *m*). Seven acoustic meta-molecules are combined to construct a cluster, and two clusters are combined to construct a cluster set. The length ratios of the units in a cluster are 1, 5/7, 4/7, 3.5/7, 3/7, 2.5/7 and 2/7; the aperture ratios in a set are 1 and 2, that is, *i* = 2 and *j* = 7.

In the Supplementary Materials, the intrinsic resonant frequencies and the double-negative frequency ranges of meta-molecules are determined based on the tube length and diameter of the side hole. Therefore, the working frequency of the metamaterial can be modulated by changing the dimensions of the meta-molecule. The locally resonant essence^35^,^37^,^38^,^39^ of the present metamaterial ensures a non-significant influence among adjacent units^30^,^31^,^32^,^33^; therefore, a broadband double-negative metamaterial can be obtained by stacking meta-molecules with different working frequencies. Simulations and experiments have revealed the number and size of the units for this model. 14 meta-molecules, which are divided into seven units, as shown in Fig. 1b, are used to construct a meta-molecule cluster set, as shown in Fig. 1c. The intervals of adjacent units along the *X*- and *Z*-axes are 1 and 2 mm, respectively. It is obvious that the physical property of our model, along the direction parallel to the tube, is different from that perpendicular to the tube; thus, the fabricated metamaterial sample shown in Fig. 1d is an anisotropy material. However, in the plane that is perpendicular to the tube, the property is the same, i.e., our model is omni-directional. In acoustics, as long as the unit size of a material along the propagation direction of a sound wave is much smaller than the working wavelength, this material can be considered as homogeneous. Although the size of the macro cell in the *X*-*Z* plane is 8 cm × 10 cm, because there is no interaction among the meta-molecules inside the macro cell, the theory of effective medium is only related to the size in the *Y*-*Z* plane and has no connection with the size along the *X*-axis. Along the *Y*-axis, the lattice constant (2.7 cm) of the sample is determined by the tube (0.7 cm in diameter) and the sponge substrate (2.0 cm in thickness). Along the *Z*-axis, there is a one-to-one correspondence between the meta-molecules with different lengths and sound waves with different wavelengths. For instance, the meta-molecule with a length of 9.8 cm corresponds to the sound wave with a wavelength of approximately 20 cm; the meta-molecule with a length of 2.9 cm corresponds to the sound wave with a wavelength of approximately 6 cm. Therefore, this type of metamaterial can be considered as a homogeneous medium and the effective parameters can then be derived from the measured transmission and reflection results^34^.

The transmission and reflection behaviours of the fabricated metamaterial sample are experimentally determined; the present cluster is also numerically simulated. The structural dimensions of the simulated cluster are similar to those of the actual sample. The experimental value is consistent with that of the simulated pattern, as shown in Fig. 1e,f. The transmittance curve shows a series of absorption peaks over a wide frequency range; within this range, phase shifts appear. This phenomenon occurs because meta-molecular units with different dimensions correspond to various locally resonant frequencies. Moreover, no interaction is observed among adjacent units; each unit can resonate independently. The peak-notch positions of the simulated and measured transmission and reflection curves have a one-to-one correspondence. The transmission valleys in the experiments slightly differ from those in the simulations because of the machining error in the preparation of practical samples. The magnitude of the experimental transmission valley diverges from that of the simulated result because the environmental parameter set in the simulation does not match the real circumstances well.

**Figure 2:**
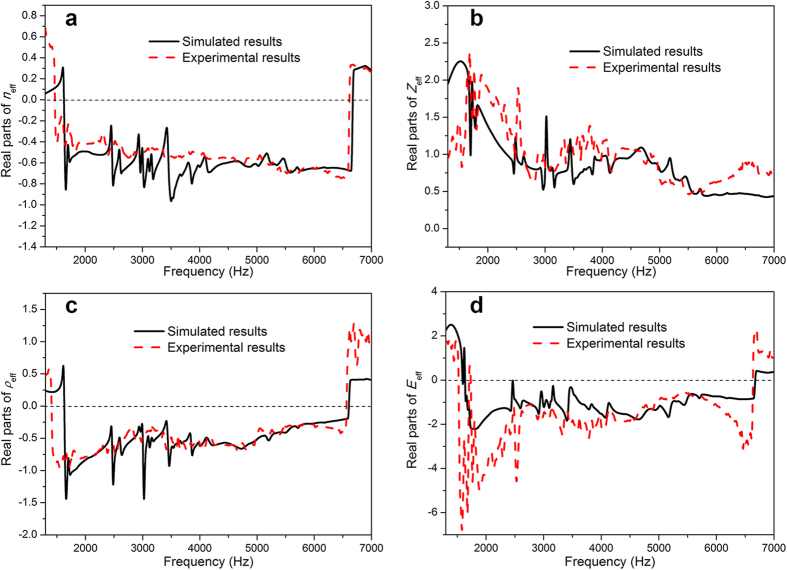
Experimental and simulated effective parameters of the metamaterial sample. (**a**) Effective refractive index. (**b**) Impedance. (**c**) Mass density. (**d**) Bulk modulus. The red and black lines represent the real parts of the results derived from the experimental and simulated data, respectively. The mass density, bulk modulus and refractive index are negative over a very wide frequency band.

Figure 2 illustrates the effective parameters, namely, refractive index, impedance, mass density and bulk modulus, as a function of frequency; these parameters are derived from the transmission and reflection results based on the retrieval method for the effective parameter^40^. The real parts of the mass density and bulk modulus of the material are negative over a broad frequency range from 1.402 kHz to 6.56 kHz and 1.74 kHz to 6.635 kHz, respectively. The real part of the refractive indices is negative from 1.472 kHz to 6.614 kHz. The deviations of derived physical parameters from the simulated results and measured results are very small: the frequency ranges in which the parameters are negative are almost exactly the same; and the amplitudes of parameters are almost identical. Because the locally resonant frequencies of all the meta-molecules in this metamaterial model are within the range of 1600 Hz to 6500 Hz, the effective parameters of the constructed cluster set are negative in this frequency range; there certainly will be abrupt changes for the parameters near 1600 Hz and 6500 Hz.

**Figure 3:**
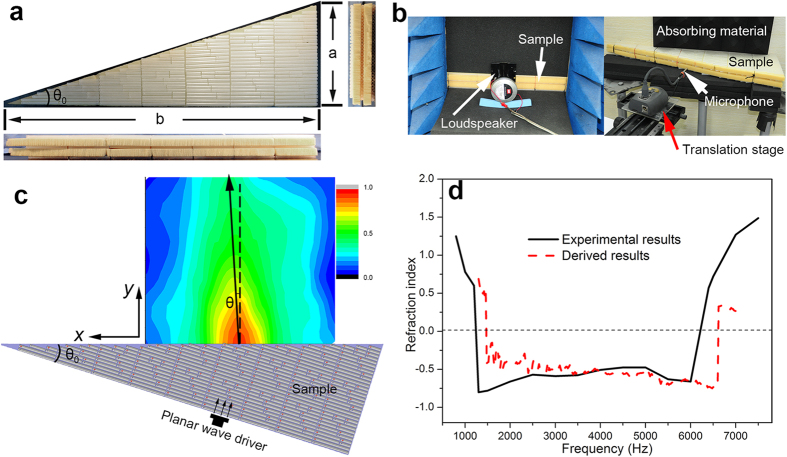
Refraction of the metamaterial sample. (**a**) Photograph of the triangular metamaterial sample; the bottom and offside sections are the lateral view of the sample, with θ_0_ = 17.5°. Lengths of the right-angle sides are a × b = 950 mm × 300 mm. **(b**) Test platform of the refraction. The left side is the acoustic emission section; the loudspeaker is a plane wave driver. The right side is the acoustic receiving section; the microphone is affixed to the translation stage. (**c**) Schematic of the measured refractive indices. The field pattern plotted in this figure is the experimental result at 3.5 kHz. (**d**) Relationship between refractive index and frequency. The black solid line indicates the measured results; the red dashed line refers to the real part of the refractive indices derived from the transmission and reflection results.

A right triangle sample is fabricated based on the meta-molecule clusters, as shown in Fig. 3a. The refraction of the sample is experimentally determined in the frequency range of 0.8 kHz to 7.5 kHz with 18 discrete frequencies. The test platform is shown in Fig. 3b. Supplementary Figure S7 reveals the detailed results. Figure 3c shows the schematic of the experimental measurement and the field distribution of sound pressure at 3.5 kHz. The transmitted and incident beams are on the same side of the normal. Based on the propagation direction of the transmitted waves, we find that the refraction of the sample is *n* = −0.577. Figure 3d illustrates the experimental and calculated results of the refractive indices of the metamaterial as a function of frequency. The measured refractive indices are negative within the frequencies between 1.238 kHz and 6.214 kHz; this result matches the calculated results well. Note that although there are differences in the direction of the propagation of sound waves between measuring the refraction and measuring the transmittance, the physical properties of the metamaterial in these directions are identical because our metamaterial is omni-directional.

**Figure 4:**
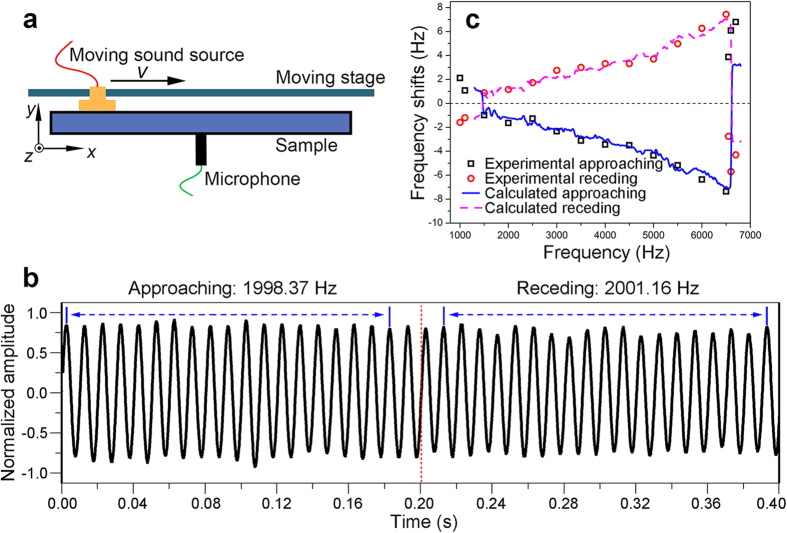
Inverse Doppler effect. (**a**) Sketch of the experimental setup of the Doppler shifts. (**b**) Oscillograms detected by a stationary microphone; the time actually needed for the moving source to pass through the entire sample is 0.8 s. A partially amplified region is shown here to facilitate understanding; the frequency of the exciting sound wave is 2.0 kHz. When the source approaches the microphone, the detected frequency is reduced by 1.6 Hz; as the source recedes, the frequency increases by 1.2 Hz. (**c**) Doppler shifts of the sample as a function of source frequency. The black boxes and red circles represent the results of the approaching and receding processes, respectively. A positive value indicates that the detected frequency is larger than the source frequency; a negative value shows that the detected frequency is less than the source frequency. The blue solid line and the purple dashed line correspond to the approaching and receding results, respectively, as derived from the refractive indices.

One of the unique characteristics of double-negative metamaterials is the inversed Doppler effect. The Doppler effect of the proposed metamaterial is experimentally investigated. The experimental setup is shown in Fig. 4a. The actual frequency of the moving source can be obtained by calculating the wave number per unit time. The Doppler shift of the constructed metamaterial can be derived based on the actual frequency. The inversed Doppler effect is experimentally investigated at frequencies ranging from 1.0 kHz to 6.7 kHz with 16 discrete frequency points. Supplementary Figure S9 shows the detailed measurement results. Figure 4b displays the signal recorded by a stationary detector at 2.0 kHz. The moving source passes the sample centre in the middle of the time axis (red dashed line); therefore, the left and right sides of the red dashed line indicate the approaching and receding processes of the moving source, respectively. The measured Doppler shifts of the broadband double-negative sample versus frequency are shown in Fig. 4c. Within a broad frequency band ranging from 1.5 kHz to 6.5 kHz, the Doppler shift of the metamaterial is abnormal.

When sound waves propagate from air to the metamaterial, the relationship between the sound velocity and refractive indices of the metamaterial is *n*_1_*v*_1_ = *n*_0_*v*_0_, where *n*_1_ and *n*_0_ are the refractive indices of the metamaterial and air, respectively; *v*_1_ and *v*_0_ represent the sound velocity of the metamaterial and air, respectively. As the observer remains motionless and the source moves, the detected Doppler shift can be derived based on the following equation^1^:


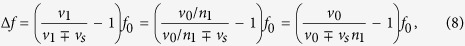


where ∆*f* is the frequency shift; *v*_*s*_ and *f*_0_ are the speed and frequency of the sound source, respectively. In Eq. (8), if the speed of the sound source *v*_*s*_ is fixed, then the Doppler shift is related only to the refractive index. The Doppler shifts calculated based on the derived refraction results are also displayed in Fig. 4c. The calculated results match the experimental results well.

**Figure 5:**
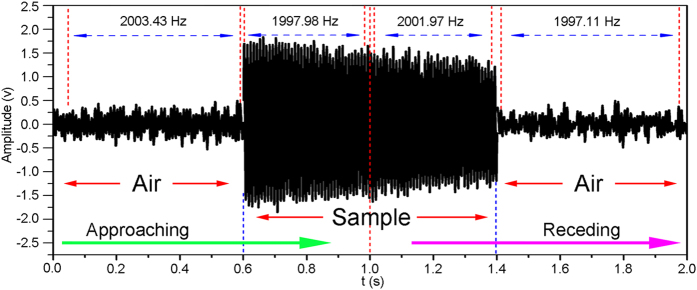
Waveform of the source frequency of 2.0 kHz during the whole moving process of the loudspeaker. The green and purple arrows refer to the approaching and receding processes of the source, respectively. The labelled frequencies are calculated for different time periods and relative movements: approaching the air, approaching the sample, receding from the sample and receding from the air.

Figure 5 illustrates the whole waveform detected by the observer, with a source frequency of 2.0 kHz, when the loudspeaker moves from one side of the 1D motorised translation stage to the other side. The moving speed of the loudspeaker is 0.5 m/s. The time required to finish the whole journal is 2 s. The lateral dimension of the sample is 40 cm and the sample is located at the centre of the stage; thus, the loudspeaker approaches the sample from 0 s to 1.0 s, recedes from the sample from 1.0 s to 2.0 s and passes through the sample from 0.6 s to 1.4 s. From 0 s to 0.6 s and from 1.4 s to 2.0 s, the loudspeaker moves completely in air; therefore, the detected signal corresponds to the sound wave propagating in the air medium. The calculated data based on the recorded waveform shows that the frequencies of the sound wave from 0 s to 0.6 s and from 1.4 s to 2.0 s are 2003.43 Hz and 1997.11 Hz, respectively, which are normal Doppler effects. By contrast, the frequencies from 0.6 s to 1.0 s and from 1.0 s to 1.4 s are 1997.98 Hz and 2001.97 Hz, respectively, which correspond to inversed Doppler effects. Therefore, the Doppler behaviour of the sound wave changes from normal to abnormal and vice versa during the whole moving process of the loudspeaker. This study is the first to demonstrate a system that simultaneously contains normal and abnormal Doppler behaviours.

**Figure 6:**
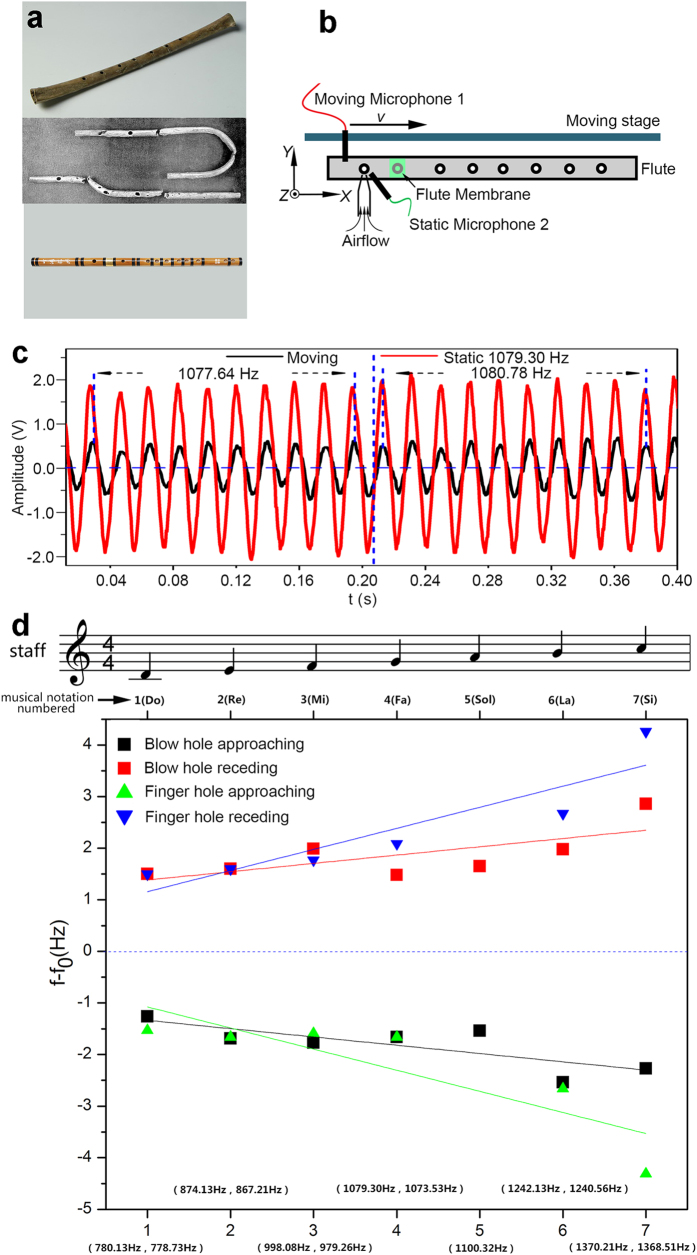
Inverse Doppler effect of the flute. (**a**) Photographs of ancient and modern flutes. (top to bottom) A bone flute excavated in Jiahu China in 1986 that has existed for 8000 years^41^; a silver flute excavated in Sumer in the Southern Mesopotamian valley in present-day Iraq in 1922 that has existed for 4500 years^44^; a modern flute. (**b**) Sketch of the experimental setup of the Doppler shifts of a flute (Supplementary Material 8. Measurements of the Doppler effect of the flute). (**c**) Oscillograms detected by the static and moving microphones of Tone 4 at the blow hole. The frequency of the source is 1079.3 Hz. When the moving microphone approaches the source, the detected frequency is reduced by 1.51 Hz; as the microphone recedes, the frequency increases by 1.26 Hz. (**d**) Doppler shifts of the flute for different numbered musical notations (i.e., 1–7). The black and red squares correspond to the frequency shift results of approaching and receding processes, respectively, which are determined near the blow hole. The blue and cyan triangles indicate the frequency shift results of the approaching and receding processes, respectively, which are determined near the first opened finger hole. The solid lines refer to the fitting lines. Positive values indicate that the detected frequency is larger than the source frequency. Negative values imply that the detected frequency is less than the source frequency.

Flutes, as one of the earliest known instruments, have existed for 8000 years^41^, or perhaps longer^42^, because of their simple construction, convenience and euphonious tone. Flute materials have evolved from ancient animal femurs to bamboo or metal in modern days; their manufacturing process has become fairly sophisticated. The tube length and opening of a flute correspond to its resonant frequency. However, limited studies have investigated the sound effects caused by the relative movement between a flute and the audience. Considering that acoustic metamaterials composed of ‘flute-like’ meta-molecules can generate inverse Doppler effects, we hypothesised that the flute also exhibits the inverse Doppler effect. We experimentally determined the Doppler behaviour of a flute using a standard acoustic test method (Supplementary Materials). Figure 6 shows the test results. The frequency detected by a moving observer exhibits an inverse Doppler behaviour compared with the frequency generated by the flute for different tones. As the observer approaches the phonation position, the received frequency decreases; conversely, as the distance between the observer and a flutist increases, the received frequency increases. The refractive indices derived from the measured Doppler shifts are negative, as shown in Table S1. Although differences in sound intensity and frequency may be observed between the blow hole and finger holes, the seven tones obtained from the blow hole and finger holes exhibit inverse Doppler effects.

The body movements of musicians are generally believed to express their emotions and catch the attention of the audience, which can be found in many videos online^43^. Our results reveal that musicians may consider the Doppler effect caused by these motions, that is, the effect of frequency shift between the source and the receiver. Flutes and several other wind instruments, such as the clarinet, oboe and suona horn, can be used to play dulcet music because the relationship between sounds created by these instruments and those received by the audience produce inverse Doppler effects. Although the Doppler effect was initially presented approximately 200 years ago, the property of the inverse Doppler effect has been utilised for thousands of years. Flutes may be considered as the oldest instruments associated with a negative refractive index in sound wave propagation; these instruments can be used as a basic unit of the design of various acoustic metamaterials.

## Conclusion

We propose a model of ‘flute-like’ acoustic meta-cluster sets and developed a broadband, omni-directional and double-negative acoustic metamaterial. The experimental results show that the metamaterials can manipulate sound waves, such as the broadband negative refraction, the inverse Doppler effect and the shift from the Doppler effect to the inverse Doppler effect, in a system consisting of a normal medium and the metamaterial. The inverse Doppler effect of the flute implies that complex broadband metamaterials can be implemented with present technologies. The acoustic meta-cluster sets provide a new way to design and fabricate metamaterials with an arbitrarily changing refractive index and broadband double-negative parameters. The method exhibits great potential in applications such as broadband absorbers and cloaking.

## Methods

### Fabrication of metamaterial samples

The lengths of the tubes in a cluster are 98, 67, 55, 48, 41, 32.5 and 29 mm. The side holes are 5 mm away from one end of the tube, with diameters of 1 and 2 mm. The external and internal diameters of the tube are 7 and 5 mm, respectively. The tubes are composed of plastic, which is sufficiently hard for acoustic waves. The propagation medium of the acoustic wave is air. The clusters are pasted periodically on the front and back surfaces of a sponge substrate to form the metamaterial sample. The thickness of the substrate is 20 mm. The sponge is a non-dispersive sound medium suitable for use as an acoustic substrate^22^. The dimensions of the constructed metamaterial sample, which is used to measure the transmission, reflection and Doppler shift, are 400 mm × 400 mm × 34 mm. The dimensions of the right-triangle sample are 950 mm × 300 mm × 51 mm, with a θ_0_ of 17.5°.

### Experimental facilities

A plane wave driver (4510ND, BMS, Hannover, Germany) is connected to a signal generator (MC3242, BSWA, Beijing, China) and a power amplifier (PA50, BSWA, Beijing, China) to generate sinusoidal acoustic waves. A free field microphone (MPA416, BSWA, Beijing, China) is connected to a lock-in amplifier (SR830, SRS, Sunnyvale, USA) to record the amplitude and phase signals of sound waves. The samples are surrounded by sound-absorbing materials to eliminate scattered waves.

### Measurement methods of the transmission and reflection of the sample

The detailed measurement methods are presented in the Supplementary Materials.

### Measurement method of the refraction of the right triangle sample

A planar wave driver is placed next to the sample. The driver then generates sound beams perpendicular to the interface. The incident beam forms an angle of 17.5° with the refraction surface. A microphone is affixed to a three-dimensional translation stage, with a scanning area of 200 mm × 50 mm, to map the sound field distribution of the refracted waves on the *X–Y* plane.

### Measurement method of the Doppler shifts of the metamaterial sample and the flute

The detailed measurement methods are presented in the Supplementary Materials.

## Additional Information

**How to cite this article**: Zhai, S. L. *et al*. Inverse Doppler Effects in Broadband Acoustic Metamaterials. *Sci. Rep.* **6**, 32388; doi: 10.1038/srep32388 (2016).

## Supplementary Material

Supplementary Information
