# Supplementary material for: Inverse Doppler Effects in Broadband Acoustic Metamaterials
Source: Sci Rep. 2016 Aug 31;6:32388. doi: 10.1038/srep32388 (PMC5006168; doi:10.1038/srep32388)
Supplement: Supplementary Information [file srep32388-s1.pdf]

Inverse Doppler Effects in Broadband Acoustic Metamaterials

S. L. Zhai, X. P. Zhao, S. Liu, F. L. Shen, L. L. Li & C. R. Luo

Smart Materials Laboratory, Department of Applied Physics, Northwestern Polytechnical University, Xi'an 710129 P. R. China. Correspondence and requests for materials should be addressed to X.P.Z. (email: xpzhao@nwpu.edu.cn).

Note 1. Relationship between meta-molecules and the cluster..2

Note 2. Imaginary parts of the effective parameters for the double-meta-molecule clusters (experimental and simulated results) 9

Note 3. Refraction of the double-meta-molecule clusters 10

Note 4. Abnormal Doppler shifts of the double-meta-molecule clusters 12

Note 5. Measurement methods of transmission and reflection 14

Note 6. Derivation of effective parameters for the metamaterials..16

Note 7. Measurements of the Doppler effect of the metamaterial sample................................17

Note 8. Doppler effect of the flute..18

Note 9. Calculations of the refractive index of the flute..22

References:..25

Note 1. Relationship between meta-molecules and the cluster

1.1 Effects of tube length on the metamaterial properties.

The effects of tube length on the metamaterial properties are investigated. Two metamaterial samples (A and B) composed of single dimension meta-molecules (with lengths of 98 and 67 mm) are manufactured. The external and internal diameters of the meta-molecule are 7 and 5 mm, respectively. The side hole of the meta-molecule is 5 mm away from one end of the tube, and the diameter of the side hole is 1 mm. The effective parameters of samples A and B are derived from the transmission and reflection results obtained by experiments and simulations[1], as shown in Supplementary Fig. S1. Supplementary Figures S1**a**2 and S1**b**2 show that the phase shifts for the samples appear at 1.7 and 2.35 kHz, which correspond to the resonant frequencies of the samples. The calculated real parts of mass density, bulk modulus, and refractive index are negative around the resonant frequencies. A comparison of the results of the two samples indicates that, as the length of the meta-molecule increases, the abnormal phenomena of the metamaterials are shifted to a low frequency. The abnormal bandwidth of the meta-molecule is also relatively narrow (i.e., the frequency fields of the negative refraction for the two samples are 1.691 kHz to 1.792 kHz and 2.367 kHz to 2.595 kHz, respectively). The deviation of the experimental results from the simulated results is due to the mismatching tolerance and experimental error.

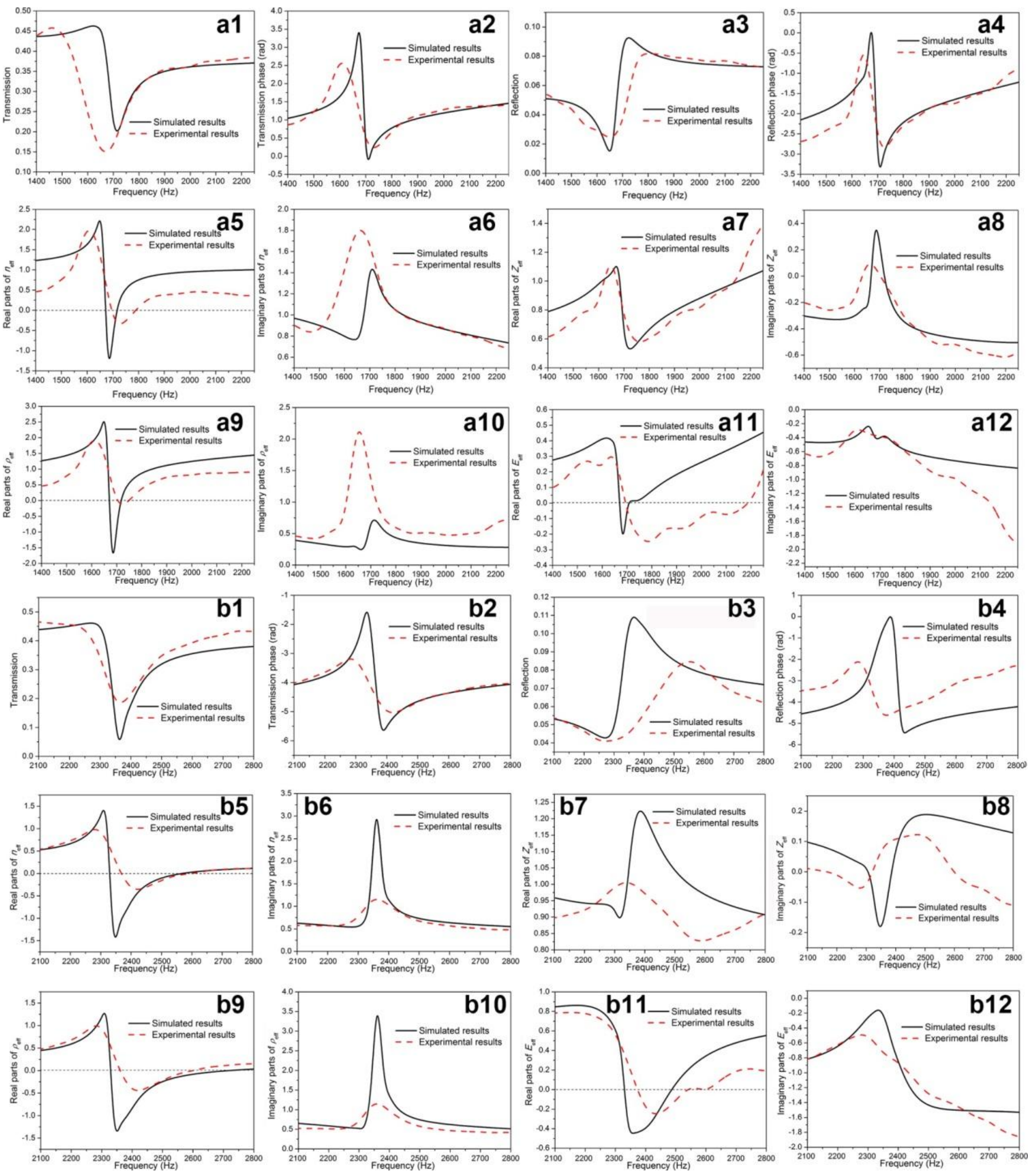

Supplementary Figure S1. Transmission, reflection, and effective parameters of meta-molecules with different lengths. The black solid lines and the red dashed lines indicate the simulated and experimental results, respectively. (**a1**)–(**a12**) represent the results of a meta-molecule with a length of 98 mm, and (**b1**)–(**b12**) refer to the results of a meta-molecule with a length of 67 mm.

Given that meta-molecules with different lengths have different double-negative frequency ranges, one of the possible methods of realizing multiband and broadband metamaterials is to

combine meta-molecules with different lengths according to a certain arrangement. Under the condition of a weak interaction among units, the double-negative frequency range of each meta-molecule can be obtained independently. Thus the double-negative region of combined meta-molecule is broadened. Based on this idea, we design a meta-molecule cluster, which is constructed by seven meta-molecules with different tube lengths (i.e., 98, 67, 55, 48, 41, 32.5, and 29 mm). The diameter of each side hole is 1 mm. The arrangement of the meta-molecules for the cluster is identical with that described in the main body of this article. The transmission and reflection results are obtained through simulations, from which the effective parameters can be derived with the results shown in Supplementary Fig. S2. The transmission phase of the metamaterial shifts at seven frequency ranges, which corresponds to the resonant frequencies of meta-molecules. The overall frequency ranges of negative mass density, bulk modulus, and refractive index are indeed broadened, but they are not connected. Therefore, the cluster constructed by seven meta-molecules with different lengths cannot realize broadband double-negative material parameters.

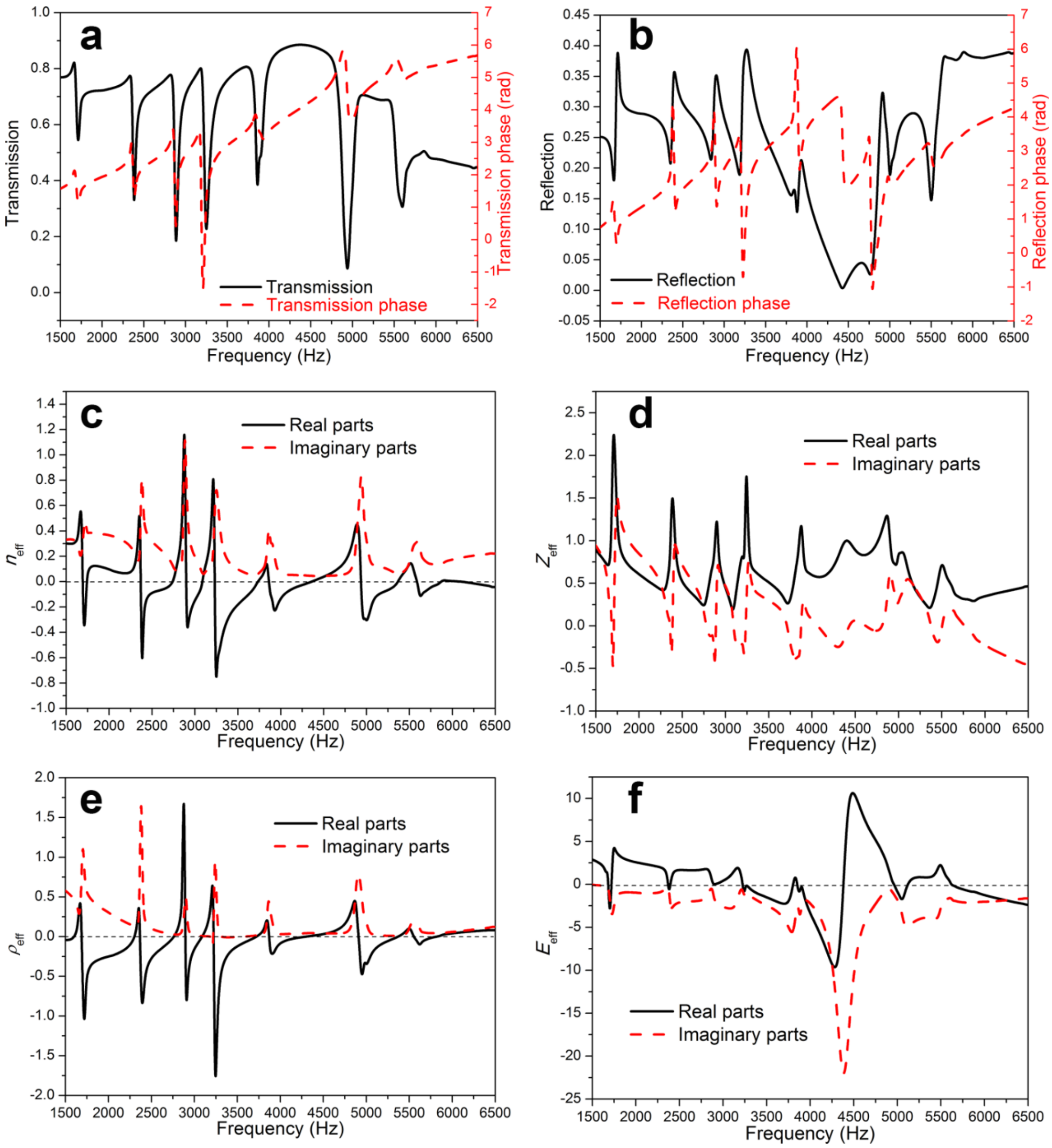

Supplementary Figure S2. Transmission, reflection, and effective parameters of a cluster composed of meta-molecules with different lengths as functions of frequencies. (**a**) Transmission and transmission phase. (**b**) Reflection and reflection phase. (**c**)–(**f**) Real and imaginary parts of the effective refractive index, impedance, mass density, and bulk modulus, respectively.

1.2 Effects of the diameter of side hole on the metamaterial properties.

In the following section, the influence of on the acoustic characteristics of a metamaterial is

studied. Three metamaterials composed of unitary meta-molecules with different configurations are simulated. The length of the meta-molecules is 67 mm, and the diameters of the side hole are 1, 2, and 3 mm for samples C, D, and E, respectively. Supplementary figure S3 displays the transmission, reflection, and effective parameters of the metamaterials. Supplementary figure S3**a**2 indicates that the frequencies of the transmission phase shifts for samples C, D, and E are 2.367, 2.464, and 2.483 kHz, respectively. These results imply that the resonant frequency of the metamaterials moves to a higher frequency as the diameter of the side hole increases. The negative refraction region also moves toward a higher frequency, as shown in Supplementary Fig. S3**c**1. Therefore, altering the hole diameter of the meta-molecule is another effective method of manipulating the abnormal frequency range of the metamaterials.

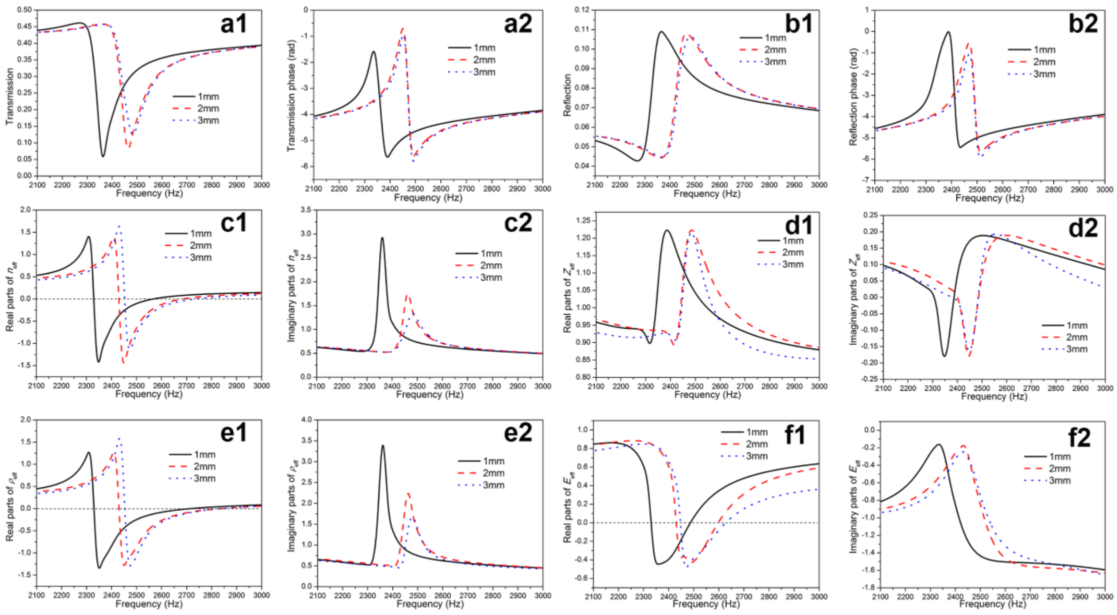

Supplementary Figure S3. Simulated transmission and reflection behavior and the material parameters for samples c, d, and e. (**a1**), (**a2**) Transmission ratio and phase, respectively. (**b1**), (**b2**) Reflection ratio and phase, respectively. (**c1**), (**c2**) Real and imaginary parts of refraction, respectively. (**d1**), (**d2**) Real and imaginary parts of impedance, respectively. (**e2**), (**e2**) Real and imaginary parts of mass density, respectively. (**f1**), (**f2**) Real and imaginary parts of bulk modulus,

respectively.

Based on the preceding discussion, we combine meta-molecules with the same length but different hole diameters to broaden the abnormal frequency range of the metamaterials. Supplementary figures S4**a**1–S4**a**8 show the simulated results of the transmission, reflection, and effective parameters of metamaterials with a tube length of 98 mm and hole diameter combinations of 1 mm and 2 mm, 1 mm and 3 mm, and 1mm and 4 mm. The transmission phase exhibits two shifts in the adjacent frequency ranges, which implies that weak interactions among meta-molecules do not significantly affect the acoustic properties of each meta-molecule. For the side holes with combined diameters of 1 and 3mm, and 1 and 4 mm, the derived negative effective parameters are indeed broadened but are not connected completely. Regarding the side hole with combined diameters of is 1 and 2 mm, the broadened negative regions are also connected. Similar results are achieved when the lengths of the meta-molecules are 67 and 41 mm.

From the preceding sections, the frequency range of the abnormal properties of metamaterials can be tuned by varying the length of meta-molecules or the diameter of side holes. Combining meta-molecules with different sizes can effectively broaden the frequency band of anomalous properties. On the one hand, although the cluster consisting of meta-molecules with seven different lengths can realize negative parameters in broad frequency bands, it cannot connect them completely. On the other hand, the combination of meta-molecules with different hole diameters cannot broaden the abnormal frequency range to a great extent, but it can connect them within a limited area. These two modulation methods are complementary.

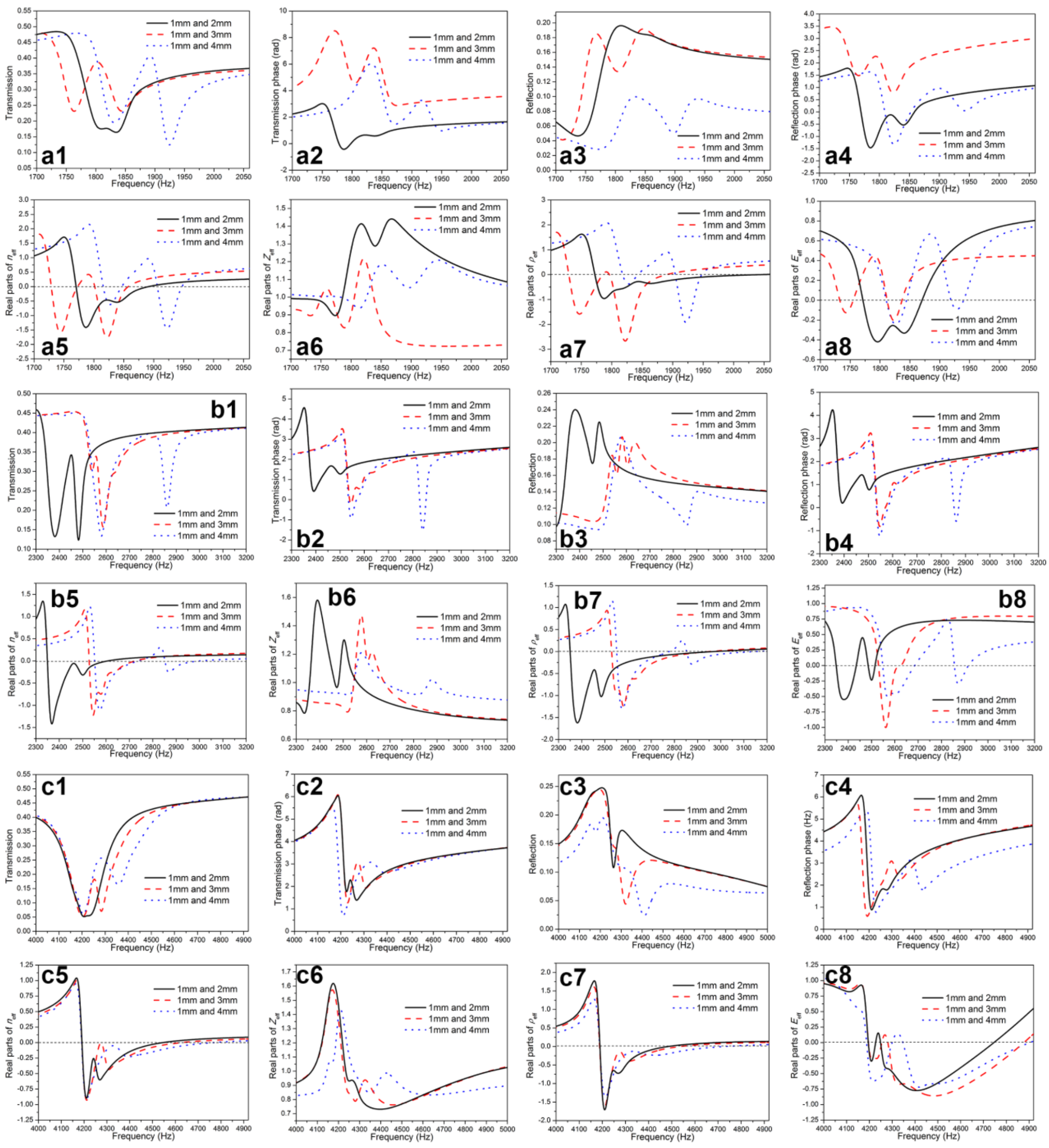

Supplementary Figure S4. Simulated material properties (i.e., transmission, reflection, refraction, impedance, mass density, and bulk modulus) of metamaterials constructed by alternatively arranging two meta-molecules with different hole diameters. (**a1**)–(**a8**) The length is 98 mm, and the side hole diameter combinations are 1 mm and 2 mm, 1 mm and 3 mm, and 1 mm and 4 mm. (**b1**)–(**b8**) The length is 67 mm. (**c1**)– (**c8**) The length is 41 mm.

1.3 Optimization design for the double-meta-molecule clusters.

The two modulation methods mentioned above are simultaneously applied to construct

meta-molecule clusters. Seven meta-molecules with different lengths are selected, each of which has two types of side hole diameters (i.e., every super-unit contains 14 meta-molecules). The lengths of the seven meta-molecules are the same as that mentioned in the main body of this paper. Three meta-molecule clusters are simulated, with hole diameter combinations of 1 and 2 mm, 1 and 3 mm, and 1 and 4 mm. Given that the first cluster has been described in the main body, Supplementary Figure S5 shows only the results for the latter two clusters. Both of the clusters cannot connect the negative frequency ranges. However, as shown in the main body of this paper, the first cluster can connect them. Therefore, the configuration of the first cluster is chosen for the design of broadband metamaterials.

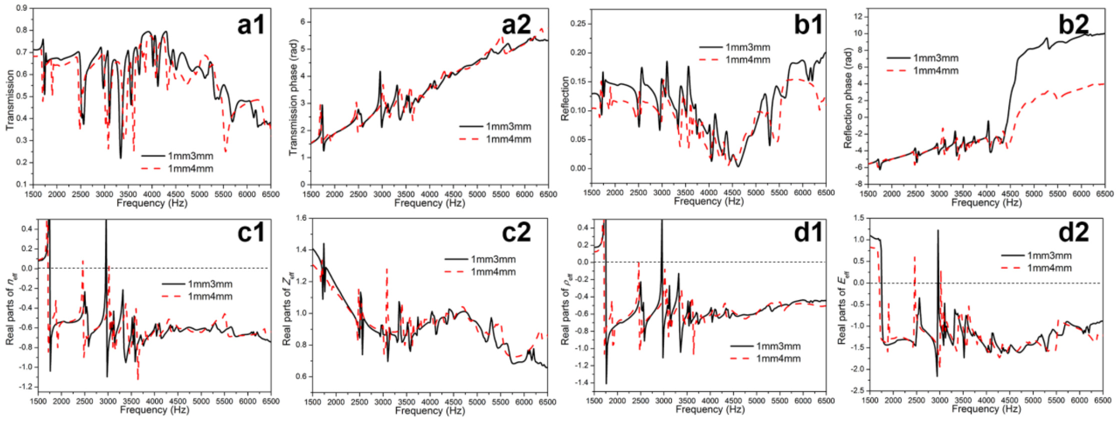

Supplementary Figure S5. Material properties of meta-molecule clusters constructed by two side hole combinations (i.e., 1 and 3 mm and 1 and 4mm). (**a1**), (**a2**) Transmission ratios and phases of clusters, respectively. (**b1**), (**b2**) Reflection ratios and phases of clusters, respectively. (**c1**), (**c2**) Real parts of refraction and impedance of clusters, respectively. (**d1**), (**d2**) Real parts of mass density and bulk modulus of clusters, respectively.

Note 2. Imaginary parts of the effective parameters for the double-meta-molecule clusters (experimental and simulated results)

The derived imaginary parts of the effective parameters of the acoustic meta-molecule cluster used in the main text are shown in Supplementary Fig. S6. The experimental results match the simulated results well.

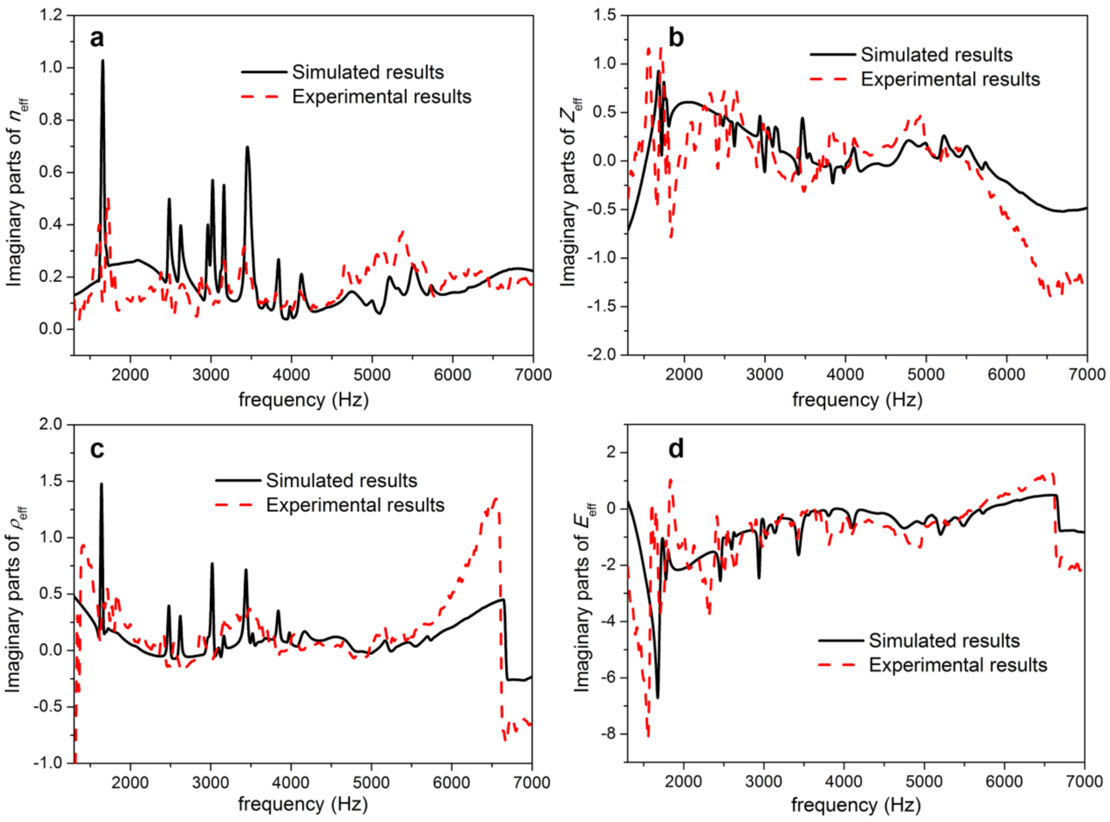

Supplementary Figure S6. Imaginary parts of the effective parameters. The black line with diamonds and the red line with dots indicate the simulated and experimental results, respectively. (**a**)–(**d**) represent the effective refractive index, impedance, mass density, and bulk modulus, respectively.

Note 3. Refraction of the double-meta-molecule clusters

Supplementary figure S7 displays the field distributions of the refracted waves from the triangular metamaterial sample at different frequencies. As the frequency increases from 0.8 kHz to 7.5 kHz, the sign of the refractive index of the fabricated metamaterial changes twice (positive–

negative–positive) [2].

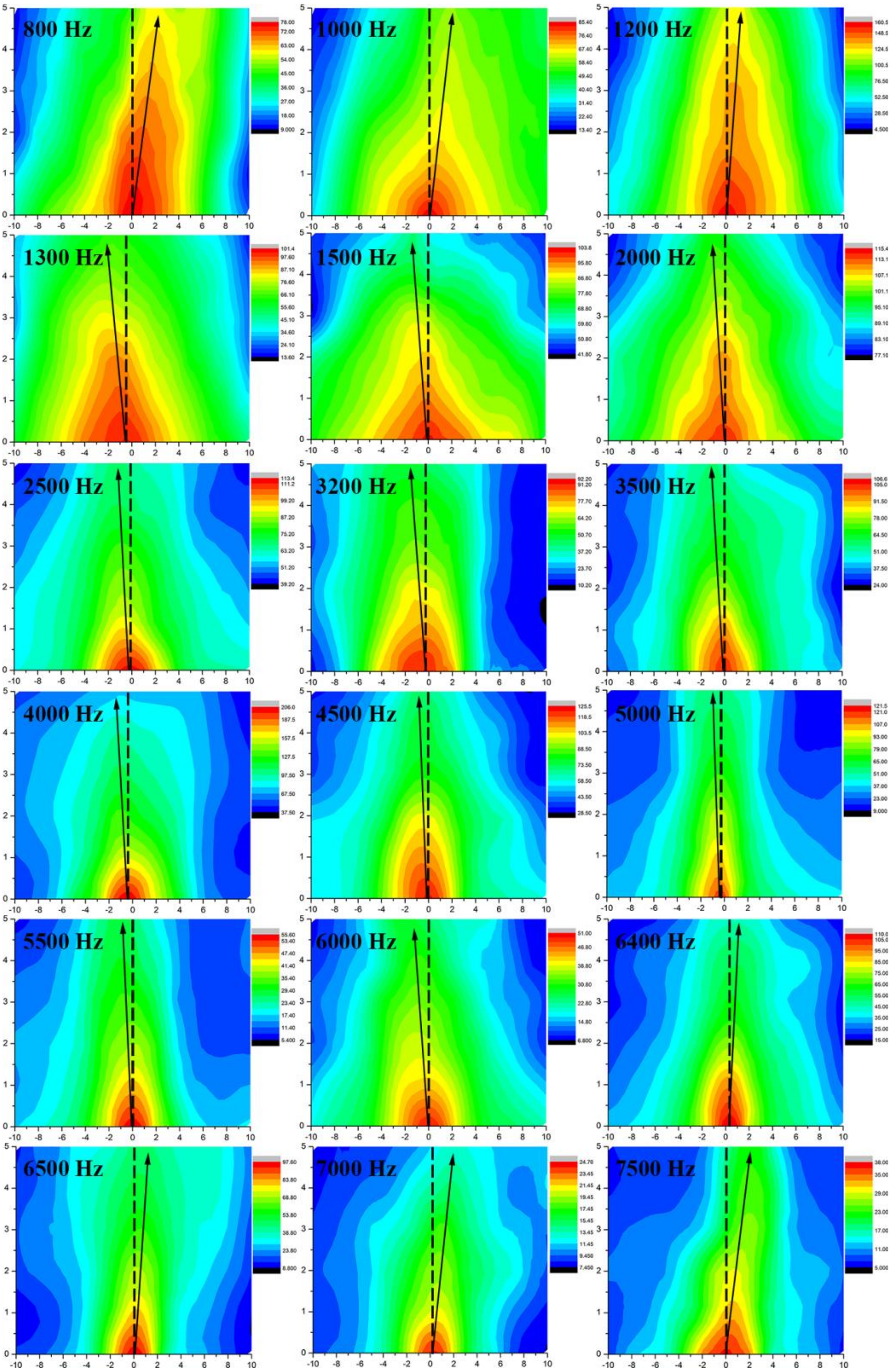

Supplementary Figure S7. Experimental results of the refraction of the metamaterial sample

at different frequencies (i.e., sound field distributions of refracted waves). The chosen frequencies are 0.8, 1.0, 1.2, 1.3, 1.5, 2.0, 2.5, 3.2, 3.5, 4.0, 4.5, 5.0, 5.5, 6.0, 6.4, 6.5, 7.0, and 7.5 kHz.

Note 4. Abnormal Doppler shifts of the double-meta-molecule clusters

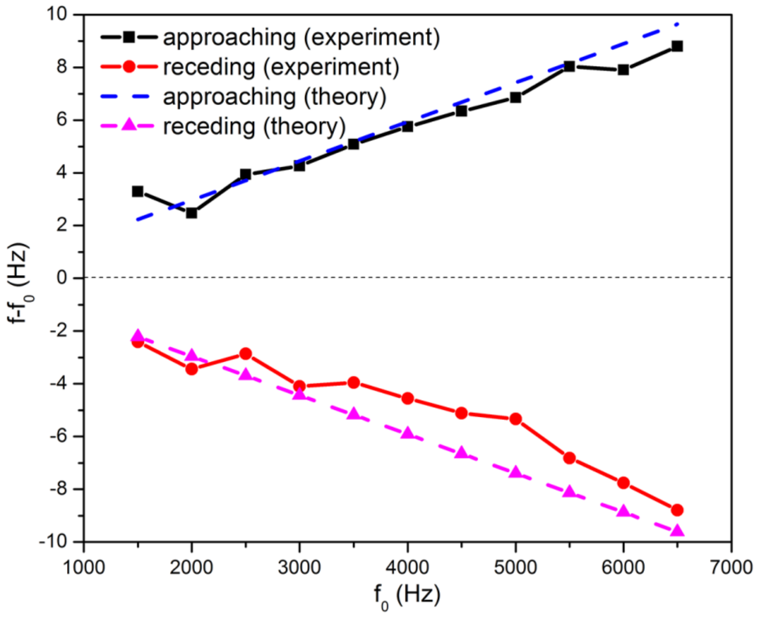

Supplementary Figure S8. Doppler shifts of air medium as a function of frequency. The solid and dashed lines indicate the experimental and theoretical results, respectively.

We measure the Doppler shift of air medium as a function of frequency using the setup described in this study. The experimental and theoretical results are shown in Supplementary Fig. S8. The experimental values match the theoretical values well. Hence, the results obtained by our experimental device are reliable[3]. The Doppler shifts of the fabricated metamaterial are also measured vis-à-vis the results in Supplementary Figure S9. The moving speed of the source is 0.5 m/s, the needed time for the loudspeaker to pass through the entire sample is 0.8s. For the source frequency f_0 = 1000 Hz, the number of the generated waveform is as much as 800 during this time.

Higher frequency will lead to more waveforms. Therefore, in order to facilitate the data processing, the recorded wave signal is compressed by using the processing function of the oscilloscope, the adjacent 20 waveforms are transformed into one waveform. The formula for calculating the frequency of the recorded signal is $f = (20 \times n) / t$, where n is the number of the calculated waveforms, and t is the needed time.

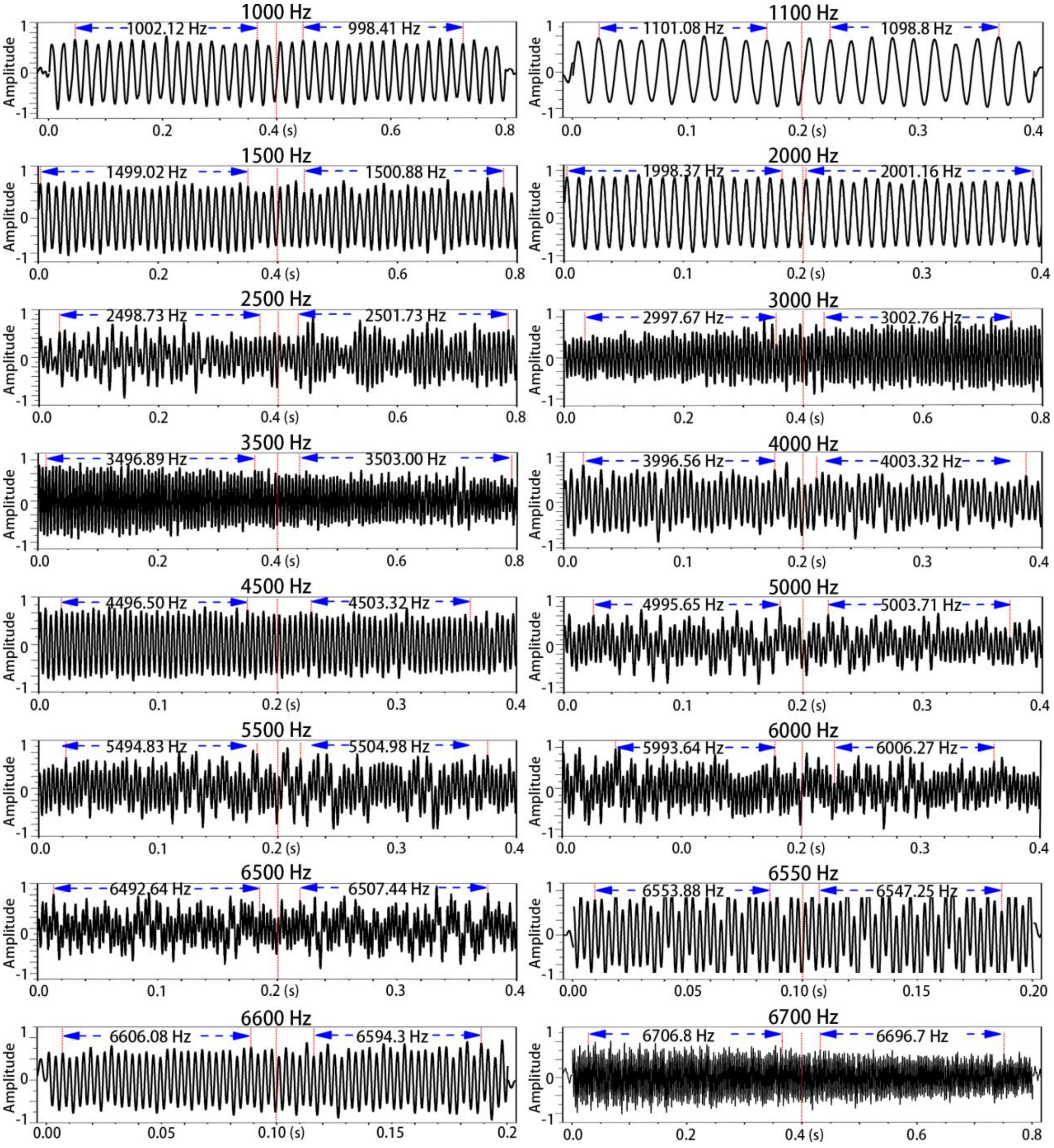

Supplementary Figure S9. Doppler shifts of the broadband metamaterial sample at different frequencies.

In summary, the resonant frequency of metamaterials can be tuned by varying the structure

size of meta-molecules. The experimental and simulated results demonstrate that the resonant frequency increases as the length of meta-molecule decreases and the hole diameter increases, but the frequency range becomes narrow. A double-meta-molecule cluster can be obtained by combining the meta-molecules with different sizes. A broadband negative acoustic metamaterial is realized using this cluster with the abnormal frequency regions of every meta-molecule cascaded. The broadband negative refraction property of this cluster is verified in the experiments, and the broadband reversed Doppler effects are experimentally achieved.

Note 5. Measurement methods of transmission and reflection

5.1 Measurement of transmission.

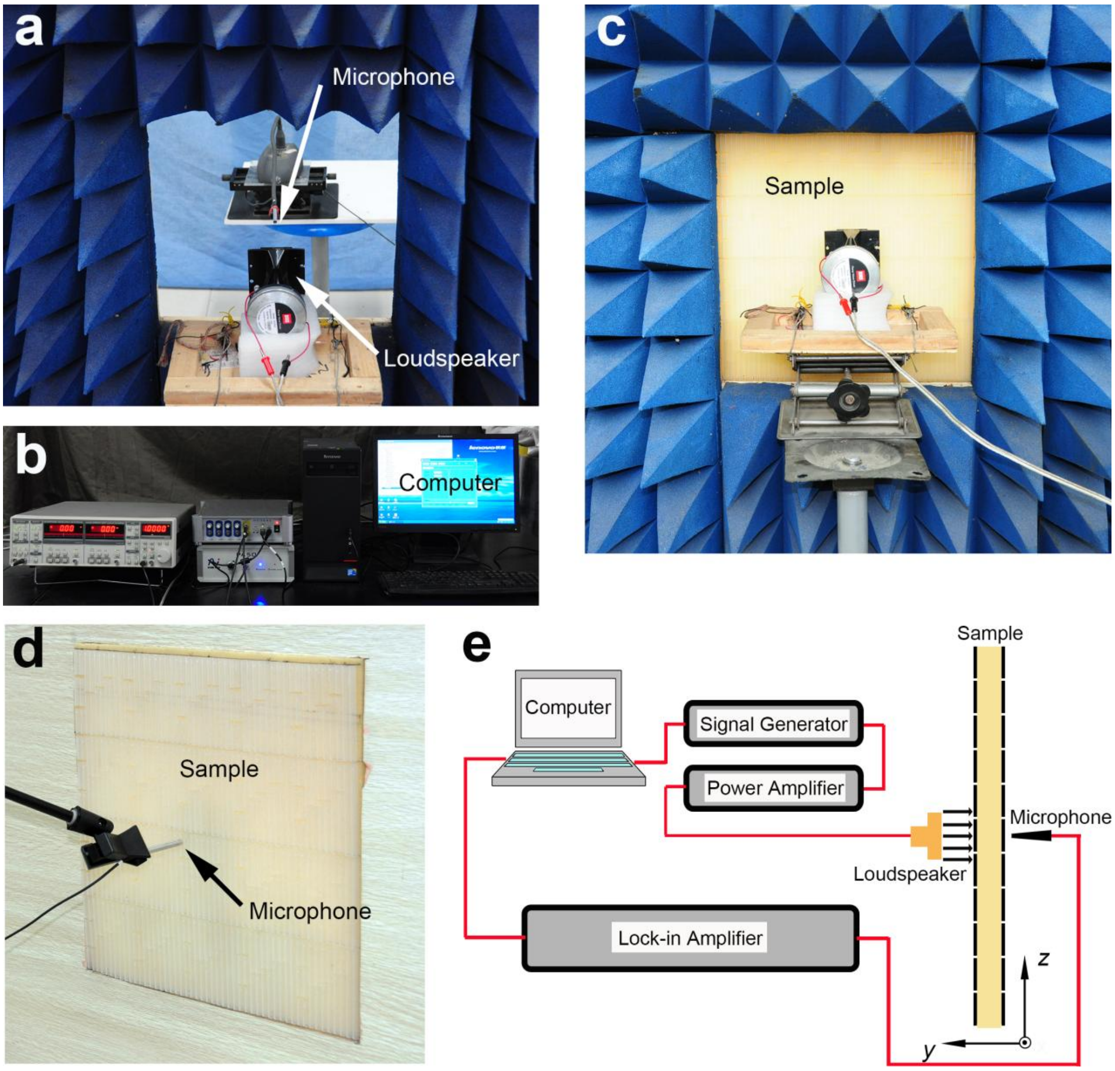

Supplementary Figure S10. Setup for the transmission. (**a**) The test platform for transmission without the sample. (**b**) Signal transmitting and receiving devices. From left to right, the apparatus is the lock-in amplifier, the signal generator and power amplifier, and a computer, respectively. (**c**) The front view of the setup. (**d**) The back view of the setup. (**e**) A schematic map of the setup.

Schematic map for the measurement of transmission is shown in Supplementary Fig. S10. A loudspeaker is located 50 mm away from the front face of the sample. Sound waves travel along the positive direction of Y-axis. After propagating through the sample, the sound waves are received by a microphone, which is 50 mm away from the rear face of the sample. Given that a lock-in amplifier can only record the amplitude and phase of sound waves with assigned frequency,

thus the interference of environmental noise is avoided. The transmitted amplitude and phase of sound waves with and without sample are A_1, φ_1 and A_0, φ_0, respectively. The transmission ratio and phase can be calculated by $T = A_1/A_0$ and $Tp = \varphi_1 - \varphi_0$, respectively[2, 4].

5.2 Measurements of reflection.

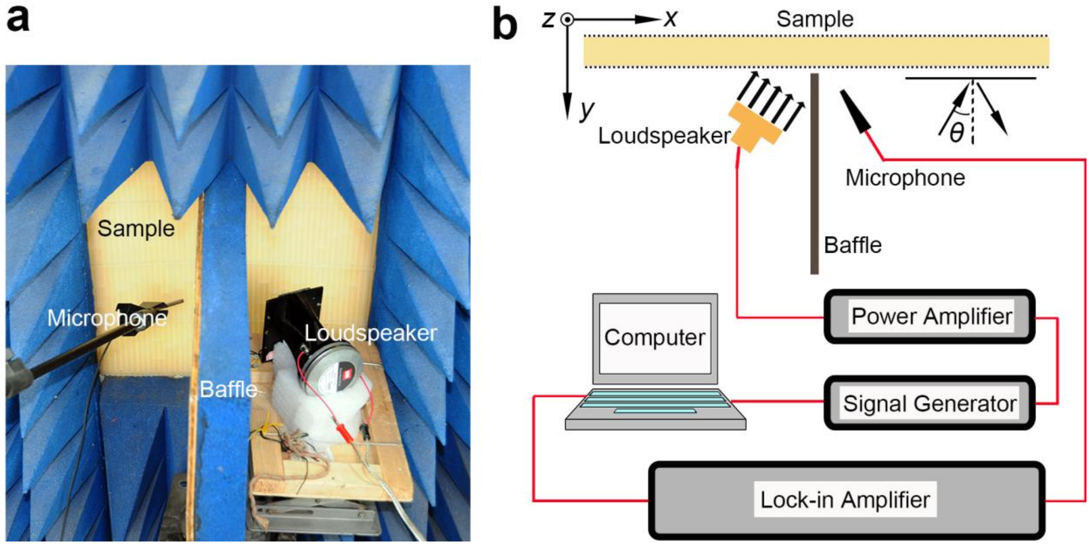

Supplementary Figure S11. setup for the reflection. (**a**) Photograph and (**b**) schematic of the setup.

The setup for measuring reflection of samples is displayed in Supplementary Fig. S11. The continuous sine waves impinges onto the sample with the incident angle of 30° , and are detected by the microphone at the other side of normal line with the same angle. The loudspeaker and the microphone are located 50 mm from the incident point. A baffle surrounded by absorbing materials is placed between the loudspeaker and microphone to avoid the incident and scattered signals to be received by the microphone. The calculations of reflection ratio and phase are similar to that of transmission.

Note 6. Derivation of effective parameters for the metamaterials

The lattice period of sample along the propagation direction of sound beam is 8 mm, whereas the shortest measuring wavelength is 48 mm, which is much larger than the lattice period. Therefore, the fabricated metamaterial can be regarded as a homogeneous medium. Based on the method of retrieving effective parameters from homogeneous mediums, the effective refractive index, impedance, mass density and bulk modulus can be calculated by the follow equations[1]:

$$n = \pm \frac{1}{kl} \cos^{-1}\left[\frac{1}{2T}\left(1 - R^2 + T^2\right) \right] + \frac{2\pi m}{kl}, \tag{1}$$

$$Z_{eff} = \pm \sqrt{\frac{(1+R)^2 - T^2}{(1-R)^2 - T^2}}, \tag{2}$$

$$\rho_{eff} = n Z_{eff} \rho_0, \tag{3}$$

$$E_{eff} = \left(Z_{eff}/n\right) E_0. \tag{4}$$

Note 7. Measurements of the Doppler effect of the metamaterial sample

The Supplementary Figure S12 shows the test platform of the Doppler effect of the sample. The sound source of sinusoidal acoustic signals is mounted on a one-dimensional motorised translation stage. The source then moves along the *X*-axis at a speed of 500 mm/s. A microphone is located at the centre of the sample to record oscillograms as the source moves from one side of the sample to the other side including the distance from the approaching and receding of sound source from the observer. The loudspeaker and microphone are near the metamaterial surface but are not in contact with each other.

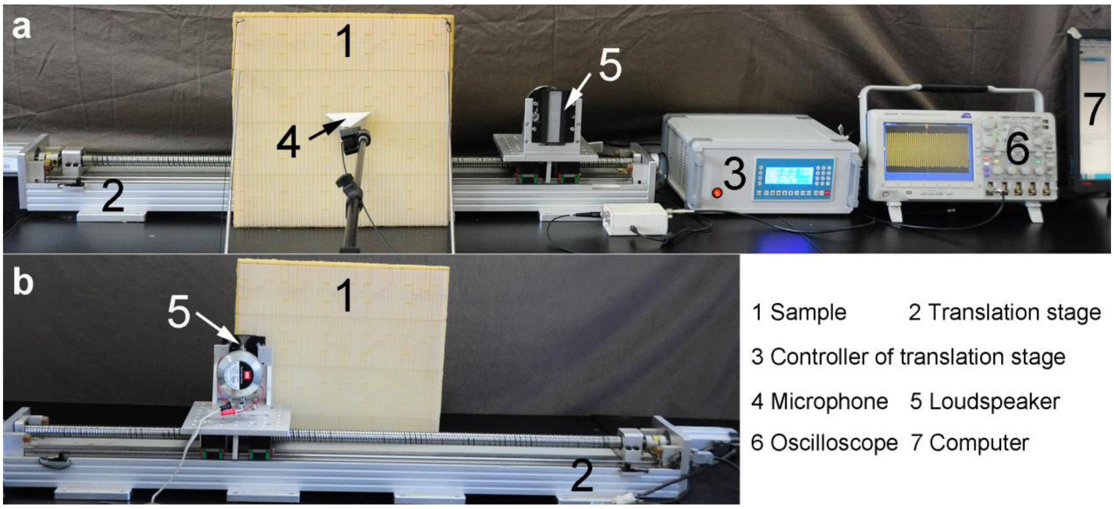

Supplementary Figure S12. Photograph of the setup for the Doppler shifts of the metamaterials sample. (**a**)The front view of the setup. (**b**) The back view of the translation stage.

Note 8. Doppler effect of the flute

8.1 Measurement method.

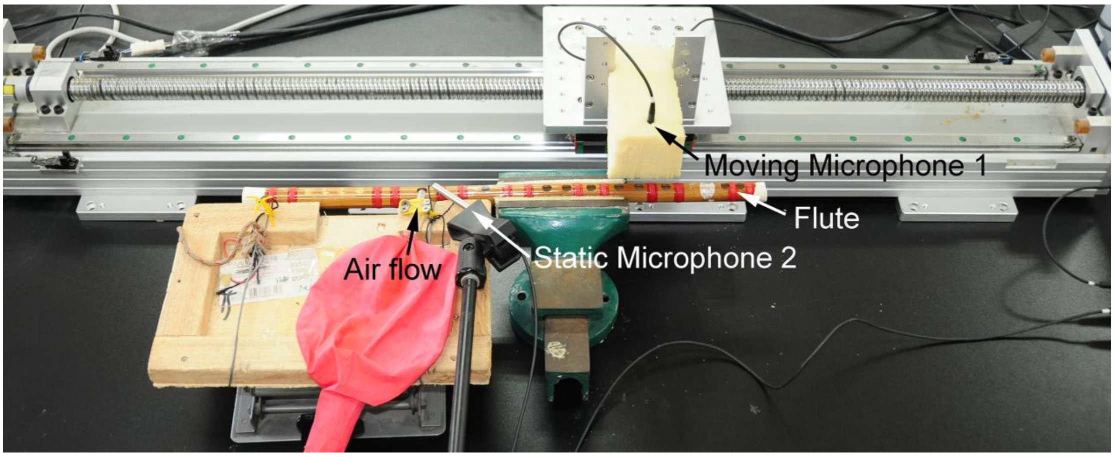

Supplementary Figure S13. Photograph of the test platform for the Doppler shifts of a flute.

The measurement of the Doppler effect of a flute is different from that of the metamaterial samples, as shown in Supplementary Fig. S13. Firstly, the device to generate acoustic waves is not a loudspeaker, but the flute itself. In order to launch acoustic waves continuously, we use a flexible pipe to connect the blow hole of the flute to a draught fan that can continue to provide a

stable air speed. Secondly, for the convenience of measurement, the position of the flute is fixed. A microphone is mounted on the 1D motorized translation stage to move toward and away from the blow hole and finger holes of the flute with a fixed speed. Finally, as the flute is driven by the draught fan, the exact stationary frequencies of the sound waves generated by the flute in different tones are unknown in advance. In order to compare the stationary frequency with the moving frequency at the same time, the second microphone is fastened near the blow hole of the flute to detect the stationary signal of sound wave at the same time as the first microphone moves toward and away from the flute. The data processing of the Doppler shifts for the flute is the same as that for the metamaterial samples mentioned above. When the speed of the moving microphone is fixed to be 0.5 m/s, the moving distance of the detector in 0.4 s is 200 mm. Using the data processing method of the metamaterial sample at the same moving speed, 20 adjacent waveforms are transformed into one waveform. The measured results of the flute in seven tones are shown in Supplementary Fig. S14. The flute exhibits inverse Doppler shifts in different tones both near the blow hole and near the finger holes.

8.2 Measurement results.

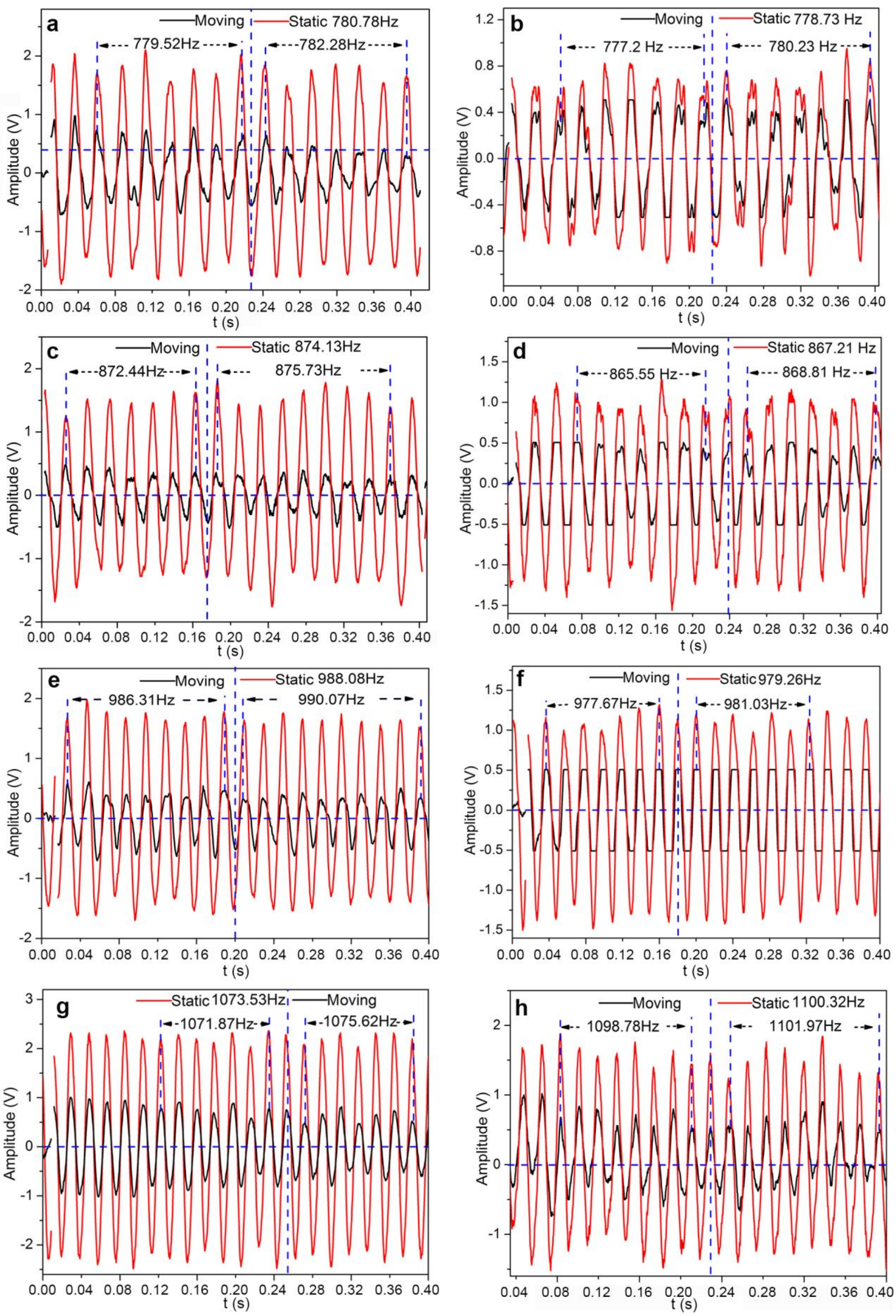

a
Moving
Static 780.78Hz
779.52Hz
782.28Hz
b
Moving
Static 778.73 Hz
777.2 Hz
780.23 Hz
c
Moving
Static 874.13Hz
872.44Hz
875.73Hz
d
Moving
Static 867.21 Hz
865.55 Hz
868.81 Hz
e
Moving
Static 988.08Hz
986.31Hz
990.07Hz
f
Moving
Static 979.26Hz
977.67Hz
981.03Hz
g
Static 1073.53Hz
Moving
1071.87Hz
1075.62Hz
h
Moving
Static 1100.32Hz
1098.78Hz
1101.97Hz
Amplitude (V)
t (s)

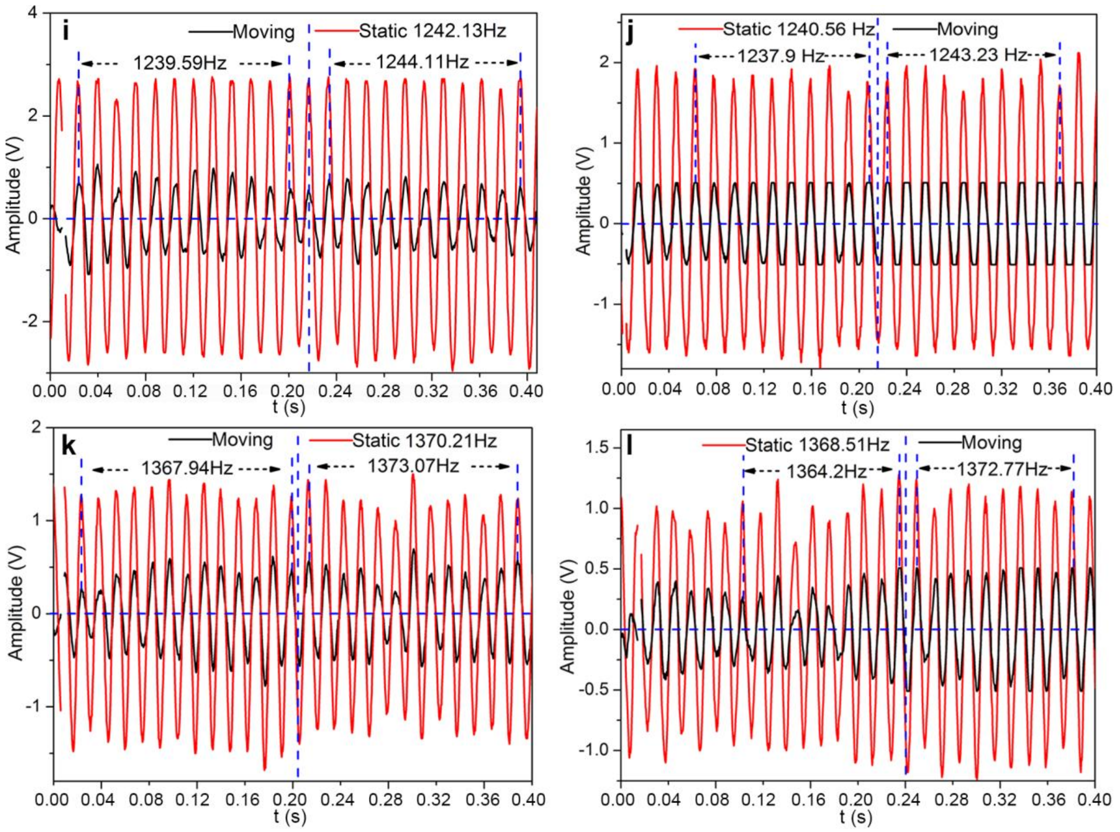

Supplementary Figure S14. Doppler shifts of the flute in different tones with the moving speed of 0.5 m/s. (**a**), (**c**), (**e**), (**h**), (**i**), (**k**) Oscillograms of sound signals detected by the two microphones at the blow hole at the tones 1, 2, 3, 5, 6, and 7, respectively. (**b**), (**d**), (**f**), (**g**), (**j**), (**l**) Oscillograms of sound signals detected by the two microphones at a finger hole at the tones 1, 2, 3, 4, 6, and 7, respectively.

We also experimentally measured the Doppler shifts of the flute at low speed at tones 2, 5, and 6, respectively. The speed of the moving microphone is 0.1 m/s, 18 adjacent waveforms are transformed into one waveform. The Supplementary Figure S15 shows the measured results. The inverse Doppler effects still exist, which demonstrates that the phenomenon of Doppler shift of the flute is independent of the moving speed of the detector.

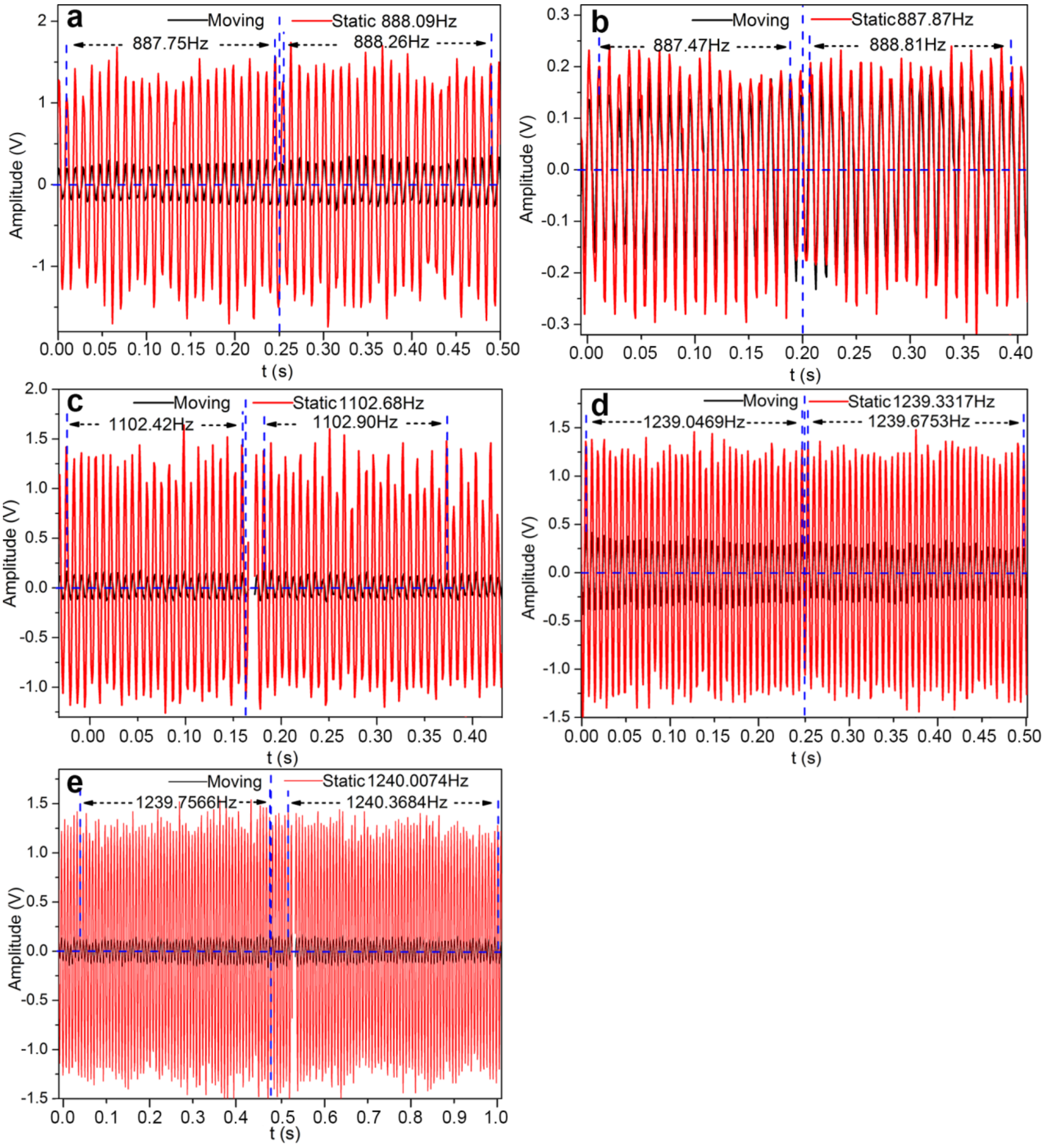

Supplementary Figure S15. Doppler shifts of the flute in different tones with the moving speed of 0.1 m/s. (**a**), (**b**) Oscillograms of sound signals detected by the two microphones at the blow hole and a finger hole at the tone 2, respectively. (**c**) Oscillograms of sound signals detected by the two microphones at the blow hole at the tone 5. (**d**), (**e**) Oscillograms of sound signals detected by the two microphones at the blow hole and a finger hole at the tone 6, respectively.

Note 9. Calculations of the refractive index of the flute

We know from the formula of Doppler shifts that, when the source is static and the observer

moves, the detected frequency shift can be expressed as $\Delta f = \left(\frac{v/n \pm v_o}{v/n} - 1 \right) f = \pm \frac{v_o n f}{v}$.

Therefore, the refractive index of the flute is $n = \pm \Delta f \cdot v/(f \cdot v_o)$, where v and v_o are the speed of sound wave in air and the speed of the moving microphone, respectively . When the microphone moves toward the flute, the sign is "+"; otherwise, the sign is "−". That is to say, if the speed of the observer is fixed, the refractive index of the flute is proportional to the Doppler shift. The refractive indexes of the flute are shown in Supplementary Table S1, it is clear that the refractive indexes of the flute are negative.

Tone		Static（Hz）	Approaching（Hz）			Receding（Hz）		
			f	Δf	n	f	Δf	n
1	Blow hole	780.78	779.52	-1.26	-1.10705	782.28	1.5	-1.31791
	Finger hole	778.73	777.2	-1.53	-1.34781	780.23	1.5	-1.32138
2	Blow hole	874.13	872.44	-1.69	-1.32628	875.73	1.6	-1.25565
	Finger hole	867.21	865.55	-1.66	-1.31313	868.81	1.6	-1.26567
3	Blow hole	988.08	986.31	-1.77	-1.22887	990.07	1.99	-1.38161
	Finger hole	979.26	977.67	-1.59	-1.11384	981.03	1.77	-1.23994
4	Blow hole	1079.30	1077.64	-1.66	-1.05509	1080.78	1.48	-0.94068
	Finger hole	1073.53	1071.87	-1.66	-1.06076	1075.62	2.09	-1.33554
5	Blow hole	1100.32	1098.78	-1.54	-0.96012	1101.97	1.65	-1.0287
6	Blow hole	1242.13	1239.59	-2.54	-1.40278	1244.11	1.98	-1.09351
	Finger hole	1240.56	1237.9	-2.66	-1.47092	1243.23	2.67	-1.47645
7	Blow hole	1370.21	1367.94	-2.27	-1.13648	1373.07	2.86	-1.43187
	Finger hole	1368.51	1364.2	-4.31	-2.1605	1372.77	4.26	-2.13543

Supplementary Table S1. The refractive indexes of the flute in different tones obtained at the blow hole and finger hole, respectively.

References:

S1. Fokin, V., Ambati, M., Sun, C. & Zhang, X. Method for retrieving effective properties of locally resonant acoustic metamaterials. *Phys. Rev. B* **76** 144302 (2007).

S2. Zhai, S. L., Chen, H. J., Ding, C. L. & Zhao, X. P. Double-negative acoustic metamaterial based on meta-molecule. *J. Phys. D: Appl. Phys*. 46, 475105 (2013).

S3. Lee, S. H., Park, C. M., Seo, Y. M. & Kim, C. K. Reversed Doppler effect in double negative metamaterials. *Phys. Rev. B* 81, 241102 (2010).

S4. Chen, H. J., Zeng, H. C., Ding, C. L., Luo, C. R. & Zhao, X. P. Double-negative acoustic metamaterial based on hollow steel tube meta-atom. *J. Appl. Phys.* 113, 104902 (2013).
